# Vector microbiota manipulation by host antibodies: the forgotten strategy to develop transmission-blocking vaccines

**DOI:** 10.1186/s13071-021-05122-5

**Published:** 2022-01-04

**Authors:** Apolline Maitre, Alejandra Wu-Chuang, Justė Aželytė, Vaidas Palinauskas, Lourdes Mateos-Hernández, Dasiel Obregon, Adnan Hodžić, Claire Valiente Moro, Agustín Estrada-Peña, Jean-Christophe Paoli, Alessandra Falchi, Alejandro Cabezas-Cruz

**Affiliations:** 1 UMR BIPAR, Laboratoire de Santé Animale, Anses, INRAE, Ecole Nationale Vétérinaire d’Alfort, 94700 Maisons-Alfort, France; 2 INRAE, UR 0045 Laboratoire de Recherches Sur Le Développement de L’Elevage (SELMET-LRDE), 20250 Corte, France; 3 EA 7310, Laboratoire de Virologie, Université de Corse, Corte, France; 4 Nature Research Centre, Akademijos 2, 09412 Vilnius, Lithuania; 5 School of Environmental Sciences, University of Guelph, Guelph, ON Canada; 6 Institute of Parasitology, Department of Pathobiology, University of Veterinary Medicine Vienna, Veterinaerplatz 1, 1210 Vienna, Austria; 7 Univ Lyon, Université Claude Bernard Lyon 1, CNRS, INRAE, VetAgro Sup, UMR Ecologie Microbienne, 69622 Villeurbanne, France; 8 Faculty of Veterinary Medicine, University of Zaragoza, Zaragoza, Spain

## Abstract

Human and animal pathogens that are transmitted by arthropods are a global concern, particularly those vectored by ticks (e.g. *Borrelia burgdorferi* and tick-borne encephalitis virus) and mosquitoes (e.g. malaria and dengue virus). Breaking the circulation of pathogens in permanent foci by controlling vectors using acaricide-based approaches is threatened by the selection of acaricide resistance in vector populations, poor management practices and relaxing of control measures. Alternative strategies that can reduce vector populations and/or vector-mediated transmission are encouraged worldwide. In recent years, it has become clear that arthropod-associated microbiota are involved in many aspects of host physiology and vector competence, prompting research into vector microbiota manipulation. Here, we review how increased knowledge of microbial ecology and vector-host interactions is driving the emergence of new concepts and tools for vector and pathogen control. We focus on the immune functions of host antibodies taken in the blood meal as they can target pathogens and microbiota bacteria within hematophagous arthropods. Anti-microbiota vaccines are presented as a tool to manipulate the vector microbiota and interfere with the development of pathogens within their vectors. Since the importance of some bacterial taxa for colonization of vector-borne pathogens is well known, the disruption of the vector microbiota by host antibodies opens the possibility to develop novel transmission-blocking vaccines.
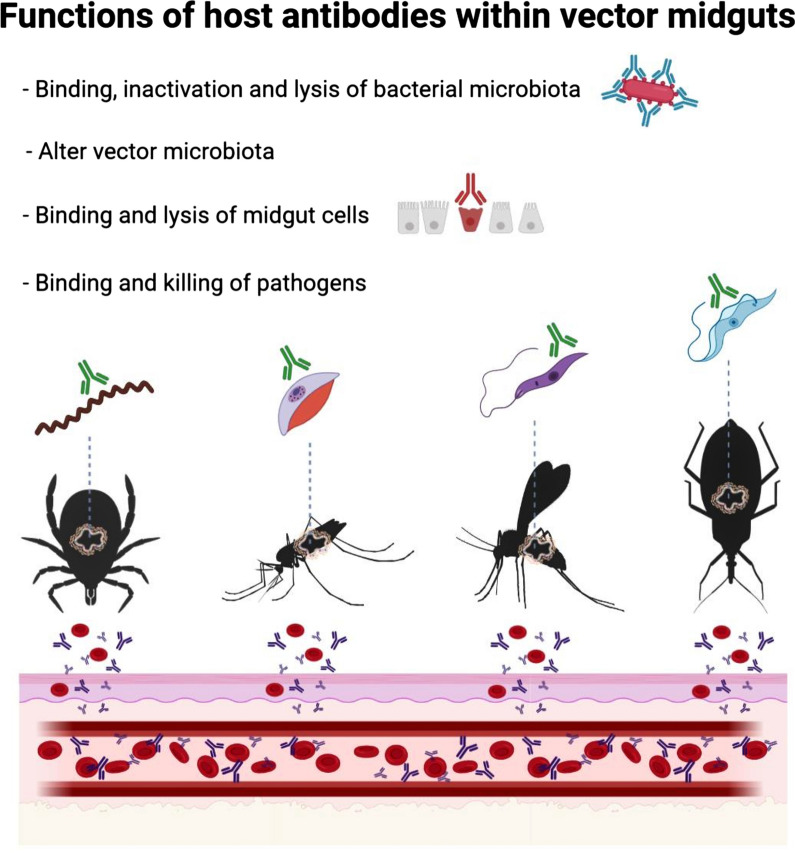

## Background

Among arthropod vectors, mosquitoes and ticks as well as sand flies and fleas are vectors of a wide spectrum of diseases with relevance in public and animal health [1–4]. For example, hard ticks (Ixodidae) transmit human and animal pathogens including bacteria (e.g. *Anaplasma phagocytophilum* and *Borrelia burgdorferi*), viruses (e.g. Crimean-Congo hemorrhagic fever virus and tick-borne encephalitis virus) and protozoa (*Babesia* spp. and *Theileria* spp.) [1]. Mosquitoes are vectors of major human diseases such as dengue (caused by dengue virus) and malaria (caused by *Plasmodium* spp.) [2]. The midgut is the first organ in which pathogenic microbes ingested with the host blood can survive and, in most cases, invade other tick [5] or mosquito [6] tissues. The midgut is also the optimal microenvironment for the survival and maintenance of the vector microbiota, likely composed of bacteria, archaea, fungi and viruses [6–8]. Within the text, “microbiome” refers to the microorganisms and their genes whereas “microbiota” only refers to the microbes themselves.

Although major emphasis has been placed on the role of endosymbionts in arthropod metabolism [9, 10] and physiology [10], the presence of multiple metabolic pathways in the microbiome of vectors such as ticks [11], mosquitoes [12] and tsetse flies [13] suggests broader metabolic complementation mediated by microbiota bacteria. Recent reports found functional redundancy (i.e. the presence of the same genes and/or functional categories in different bacterial species within a microbial community) as a property of the tick microbiome [14, 15]. Taxonomic and functional composition analyses revealed that the microbial diversity of the tick microbiome varies according to different factors such as tick species, sex and environmental conditions among others [8, 15]. The contribution of symbionts to vector fitness has been demonstrated. For example, the symbiont *Wigglesworthia* supplies tsetse flies with B6 vitamin, which, along with folates and thiamine, is necessary for the physiology and reproduction of these flies [13]. In *Aedes aegypti* mosquitoes, B vitamins can be provided by *Escherichia coli* [12]. The lack of these vitamins has been associated with developmental atrophies in the larval stages of mosquitoes [16]. Of special interest are the interactions between the vector, its microbiota and transmitted pathogens since commensal bacteria interact with vector-borne pathogens [8, 17] and can facilitate [18] or compete [19] with pathogen colonization and development within the vector midguts, prompting research into microbiota manipulation for blocking pathogen transmission [20].

Antibiotics are commonly used in microbiota manipulation studies [21–23]. Using antibiotics for microbiota manipulation is not a viable alternative to block pathogen transmission because of the increase in bacterial strains with antibiotic resistance that affects human and animal health. In addition, the effect of antibiotics on the microbiota is not specific, as several bacterial species can be depleted by antimicrobial treatment. Despite recent advances in vector microbiota research, the lack of tools for the precise and selective manipulation of the vector microbiome is currently a major limitation to achieving mechanistic insights into pathogen-microbiome interactions [20, 24]. Recently, our team introduced anti-microbiota vaccines [25] as an innovative approach to vector microbiome manipulation [26] and the development of novel pathogen transmission-blocking vaccines [27]. Host immunization with keystone taxa (i.e. highly connected taxa driving community composition and function) identified in the tick microbiota elicited bacterial-specific antibodies that caused high mortality in feeding ticks [25]. Tick mortality was associated with a host antibody response against the carbohydrate Galα1-3Gal (α-Gal), a product of galactosyltransferase enzymes with genes widely present in the tick microbiota [25]. Anti-microbiota vaccines [25, 26] can be used as a tool to induce bacterial-specific antibodies for microbiota manipulation and pathogen control. In this context, understanding the dynamics and activity of host antibodies within the vector becomes an important research area. Here we review how increased knowledge about multipartite interactions among pathogen, vector, microbiota and vertebrate hosts is driving the emergence of new concepts and tools for vector-borne pathogen control. We then focus on the dynamics of host antibodies and their interaction with pathogens and commensal bacteria within vector arthropods as an alternative for taxon-specific manipulation of the microbiota. Although the review is mainly focused on ticks, examples from other vectors are also documented.

## Vector-pathogen-microbiota interactions, a source of new targets for pathogen control

Recent research on vector-pathogen-microbiota interactions shows that microbial communities within vectors strongly influence pathogen colonization and transmission [8]. For example, tick microbiota composition influences *B. burgdorferi* colonization within the tick vector [28], and infection by the obligate intracellular bacterium *A. phagocytophilum* modulates the tick microbiota [29, 30]. By rearing *Ixodes scapularis* ticks in a sterile environment from egg to adult tick development, the ticks showed a decrease in abundance of bacteria of the genera *Acinetobacter*, *Brevibacterium*, *Lysinibacillus* and *Staphylococcus* compared to ticks grown under non-sterile conditions in the laboratory [28]. Ticks raised in sterile conditions also had a decrease in *B. burgdorferi* colonization after feeding on an infected mouse, suggesting that the composition of the microbiota alters *B. burgdoferi* infection [28]. The presence of *A. phagocytophilum* in the guts of *I. scapularis* induces the expression of the antifreeze protein IAFGP, which decreases the occurrence of the polysaccharide biosynthesis pathways involved in biofilm formation in the tick microbiome [15] and inhibits the formation of biofilms by gram-positive bacteria such as *Enterorocci* [31]. Further studies showed that the presence of IAFGP facilitates the infection of *A. phagocytophilum* in *I. scapularis* ticks [30]. Two recent epidemiological studies also revealed significant associations between the persistence of *B. burgdorferi* and the occurrence of specific microbial taxa in *I. scapularis* microbiota [32, 33]. These results suggest that *B. burgdorferi* requires a specific gut microbial composition for successful pathogen colonization in the vector. In addition, Gall et al. [23] demonstrated that microbiota disruption with antibiotics affects the acquisition of the pathogen *Anaplasma marginale* in the vector *Dermacentor andersoni*. Furthermore, although the nature of the relationship between pathogen co-infection and vector microbiota composition remains unclear, empirical work suggests that, for example, *A. phagocytophilum* and *B. burgdorferi* interactions can be mediated by the tick vector and its microbiota (revised in [8]). Tick microbiota is, therefore, very sensitive to the acquisition of new pathogens, and the direct modulation of microbe-microbe interactions can serve as a weapon against the pathogen and the effectiveness of the tick as a vector.

Similar findings in mosquitoes suggest that microbiota manipulation may cause harm to the vector and interfere with vector-borne pathogen infectious cycles [20]. The gut microbiota has been regarded as an important player in defense mechanisms against pathogens in several mosquito species, which are vectors of epidemiologically important pathogens such as, for example, *Anopheles* mosquito vectors of human malaria, *Culex* species as vectors of avian malaria and West Nile and *Aedes* species, which transmit avian malaria, and the viruses chikungunya, dengue, Zika and yellow fever [34]. Gram-negative bacteria have been shown to have the most associations with the *Plasmodium* parasite while gram-positive bacteria had no prominent effect on the development of malaria infection [35, 36]. Some species of *Enterobacter, Escherichia, Serratia* and *Pseudomonas*, commonly found in *Anopheles* mosquitoes, can markedly reduce intensities and prevalence of human and rodent malaria infection [36]. The bacterium *Asaia bogorensis* remodels glucose metabolism in a way that increases midgut pH, thereby promoting *Plasmodium berghei* (the agent of rodent malaria) gametogenesis within *Anopheles stephensi* [18], while *Aedes* mosquitoes positive for *Serratia marcescens* were more permissive to dengue virus infection [37]. The microbial communities of mosquito midgut have been shown to activate mosquito immune defense response to pathogen colonization [38–40]. It was previously thought that gut bacteria have no direct interactions with *Plasmodium* parasites and can influence pathogen colonization only through the immune defense system of mosquitoes. However, Cirimotich et al.'s [36] study showed that *Enterobacter* bacteria can produce a short-lived anti-*Plasmodium* molecule, like reactive oxygen species (ROS), which in high concentrations can significantly reduce *P. berghei* intensities in vitro.

Mounting evidence suggests that the contributions of the vector microbiota to vector physiology and pathogen lifecycle are so relevant that biology and vectorial capacity cannot be understood without considering microbial communities within the vectors. The evidence suggests that microbiome manipulation can be used to disrupt and/or block the pathogen life cycle within the vector. Indeed, several strategies for microbiome manipulation are used as a means for blocking transmission [20]. Among the most utilized strategies are the identification of naturally occurring microorganisms that impair pathogen fitness [19, 41], the design and development of paratransgenic bacteria [42] and the dissemination of microorganisms such as *Wolbachia* that are naturally spread from mother to offspring and can block the development of some pathogens [43, 44]. Studies on human malaria showed that the clearance of microbiota with antibiotic treatment can significantly enhance mosquito susceptibility to the pathogen [38, 45]. Furthermore, vector microbiota disturbance by exposure to penicillin-streptomycin reduced Arbovirus infection in *Ae. aegypti* [37] while enhancing the susceptibility of *Anopheles gambiae* mosquitoes to *Plasmodium falciparum* infection [21]. Notably, the effects of azithromycin and doxycycline on the mosquito microbiota produced differential alteration in the vectorial capacity of human malaria mosquitoes, as azithromycin decreased *P. falciparum* load and, at high concentrations, doxycycline increased *P. falciparum* infection load [22]. Mosquito microbiota can also be easily disrupted by the introduction of extrinsic bacteria, which influence pathogen development and transmission [35]. For example, experimental transference of the intracellular bacterial endosymbiont *Wolbachia* to *Ae. aegypti* inhibits the ability of chikungunya and dengue viruses and of the avian malaria agent *Plasmodium gallinaceum* to infect mosquitoes [46, 47]. In natural conditions, these bacteria are not frequently found in *Ae. aegypti*, but are frequently found in *Aedes albopictus* [46]. This suggests that introduction of uncommon members of the microbiome can disrupt potential co-evolution between pathogens and the microbiota. The genus *Wolbachia* is formed by a large group of intracellular bacteria that have been extensively used in several medical and veterinary applications [48]. An additional study revealed that resistance to Zika virus infection in *Ae. aegypti* mosquitoes was associated with the presence of *Wolbachia* in the vectors [49]. Salivary glands of mosquitoes harboring *Wolbachia* did not contain any infectious virus [49]. *Wolbachia*'s ability to spread through insect populations and impact vector capacity makes it a good tool to study pathogen transmission with high potential for the control of vector-borne diseases [48]. However, the mechanisms underlying the caused effects are not fully understood [34]. In addition, strains of *S. marcescens* were found to impact the establishment of the parasite *Trypanosoma cruzi* in the vector *Rhodnius prolixus* [41] and the *Anopheles* mosquito's capacity for *Plasmodium* transmission [19]. The *R. prolixus* symbiont *R. rhodnii* loaded with anti-microbial peptides as a paratransgenic system effectively killed *T. cruzi* parasites [42]. For a detailed revision of current strategies used for insect microbiome manipulation and blocking pathogen transmission, the reader is referred elsewhere [20]. Surprisingly, host antibodies specific to bacterial microbiota have been barely used for microbiome manipulation and transmission blocking strategies.

## Dynamics of host antibodies within hematophagous ectoparasites

**Fig. 1:**
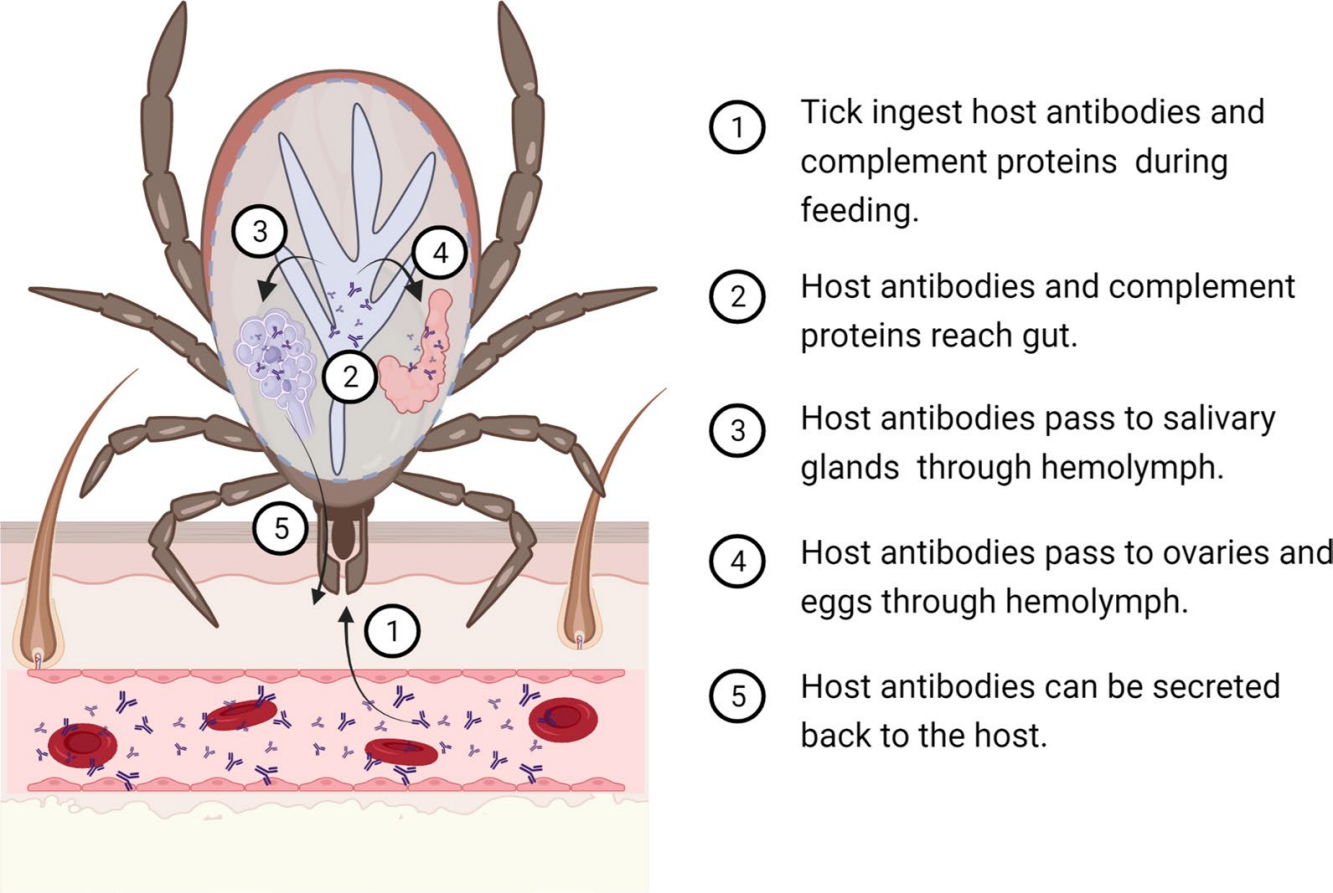
Dynamics of host antibodies within ticks. The tick midgut is the first body organ in contact with host immune components present in the blood. After crossing the gut barrier, host antibodies and complement proteins can reach the tick hemolymph and access the tick ovaries, eggs and salivary glands. Created with BioRender.com

Ticks ingest large amounts of blood from the vertebrate host during feeding. The tick midgut is the first body organ in contact with host immune components present in the blood. After crossing the gut barrier [50–52], host antibodies [53] and complement proteins [54] can reach the tick hemolymph [50–52] and access the tick ovaries and eggs [55] as well as salivary glands and be secreted back to the host [52] (Fig. 1). Host immunoglobulin (Ig) G (IgG) persisted through metamorphosis to the nymphal and adult stages of *Dermacentor variabilis* and *I. scapularis* ticks, although after molting; host IgG levels declined considerably faster in *I. scapularis* compared with *D. variabilis* [56]. In both tick species, the crossing of host IgG from the midgut into the hemocoel occurred during the later phases of engorgement [56]. Notably, the immune functions of antibodies and complement are retained in the tick tissues [50–52]. For example, intact host C3 was found in the blood meal, and full-length and cleaved C3s were observed within *I. scapularis* nymphs [54]. The IgG found in the hemolymph of the soft tick *Ornithodoros moubata* was shown to have the same antibody activity as ingested IgG [57]. Active IgG can last long periods of time within the tick. The IgG titer and activity reached a maximum at 7 days post-engorgement and remained high for > 4 months during and after oviposition in *O. moubata* [57].

Host antibodies and/or complement proteins have also been detected in the guts of other hematophagous ectoparasites such as mosquitoes [58, 59], sandflies [60, 61] and tsetse flies [62]. For example, mouse antibodies were found to persist for 2–3 days after the blood meal in the mosquito *Ae. aegypti* [58]. After ingestion, the antibodies were bound to the cytoplasm and the microvilli of mosquito midgut epithelial cells [58]. However, rat antibodies were undetectable in the same mosquito species [63], suggesting that host species may influence the persistence of antibodies within mosquitoes. Another study tracked the fate of host antibodies by feeding *An. stephensi* mosquitoes with sheep blood mixed with antibodies specific to bovine serum albumin (BSA) [59]. The anti-BSA antibody concentration at 24 h was directly related to that fed to the mosquitoes during artificial feeding, and homogenates of mosquito bodies excluding the intact guts were always antibody-positive up to 9 days post-feeding [59]. Undigested anti-BSA antibodies were also detected in the mosquito hemolymph [59], suggesting that as in ticks, host antibodies have broad access to mosquito tissues. Rat complement components necessary to initiate the alternative pathway (factor B, factor D and C3) as well as C5 were also present in the mosquito midgut for several hours following blood ingestion [64].

When feeding on human blood, the hemolymph of tsetse flies contains human albumin. Ingestion of albumin-specific antibodies was found to deplete the human albumin, which was associated with damaged osmoregulation and high mortality in these flies [62]. This shows that host antibodies ingested by tsetse flies remain functional and can affect vector fitness by depleting diet proteins. Host immune proteins such as IgG, IgM and the fraction C3 of the complement system were found to persist in the sandfly *Phlebotomus papatasi* for longer than host albumin, which disappeared rapidly, suggesting that, within the vectors, host immune proteins are relatively resistant to proteolytic degradation compared to other serum proteins [60]. The functionality of host immune proteins was demonstrated in the vector *Lutzomyia longipalpis* in which the midgut epithelium was found to activate the alternative, classical and lectin pathways of the human complement system as well as the antibody-independent C1 deposition mechanism [61].

Once in the vector’s midgut, host antibodies interact not only with tissues and surface proteins [53, 65], but can also be specifically transported inside the cells where they can interact with intracellular proteins [66–68]. Targeting vector proteins with host antibodies is the rationale behind using vaccines for the control of vector arthropods such as ticks [53] and mosquitoes [69]. For example, host antibodies against the protective tick antigen Bm86, a glycoprotein predominantly located in the membrane of tick gut cells [65], bind to the surface of epithelial cells in the tick intestine [53] causing cell lysis and reducing reproductive efficiency of engorged females [53]. Likewise, purified IgG targeting the extracellular domain of glutamate-gated chloride channel from *A. gambiae*, also a transmembrane protein, reduced the mosquito survival in a dose-dependent manner [69]. Intracellular proteins such as P0 [67, 68], involved in the assembly of the 60S ribosomal subunit, and the transcriptional factor Subolesin [70] were shown to be good targets of anti-tick vaccines. These results indicate that host immune components present in the blood not only access the vector tissues, cellular membranes and intracellular space, but are also functional after blood ingestion.

## Interaction between host antibodies and vector-borne pathogens within vectors

Once ingested, host immune components can remain active from a few hours to months depending on the species of blood-sucking arthropod, raising the possibility that vertebrate antibodies could interact with pathogens and microbiota. Empirical work shows that host antibodies can target vector-borne pathogens within ticks [71] and mosquitoes [72–74]. Targeting pathogen proteins expressed within the arthropod vectors is the rationale behind transmission-blocking vaccines [73–75]. For example, Kumar and colleagues [71] identified BBA52 as an outer membrane surface-exposed protein expressed preferentially by *B. burgdorferi* in the feeding tick. Passive transfer of anti-BBA52 antibodies into the guts of *B. burgdorferi*-infected ticks did not affect bacterial burdens within the guts of unfed or fed nymphs, but blocked spirochete transmission to the murine hosts [71]. The results suggested that the anti-BBA52 antibody blocks spirochete transmission by binding to BBA52 and interfering with protein function rather than triggering a bactericidal mechanism [71]. Likewise, it was shown that the activity of antibodies against OspA, another transmission-blocking Lyme disease vaccine target [75], does not require bacterial killing [76]. Further studies showed that host complement did not contribute to protection from nymph to host transmission because an OspA monoclonal antibody was equally effective whether infected ticks fed on normal or complement-deficient mice [54, 76]. Intriguingly, host complement enhanced the ability of anti-OspA antibodies to block tick larvae from acquiring spirochetes from mice hyperimmunized with OspA [54].

Several proteins expressed by *P. falciparum* mosquito stages have been identified [73, 74]. Three of them, Pfs48/45, Pfs230 and Pfs25, are currently targeted as lead candidates for transmission-blocking vaccines [73, 74]. Antibodies to Pfs230-C, Pfs25 and Pfs48/45 proteins elicited by vaccination effectively suppress both oocyst burden and percentage of mosquitoes infected by *P. falciparum* gametocytes in *Anopheles* mosquitoes [73, 74]. As in tick-borne pathogens, transmission-blocking vaccines against mosquito-borne pathogens such as *Plasmodium* sp. are generally accepted to act by inducing antibodies that interfere with the biological function of accessible parasite surface molecules in the mosquito midgut [77]. Notably, antibodies against Pfs230C reduced the number of *Plasmodium* oocysts > 80% in the presence of active complement and < 40% in the absence of complement [72]. These results suggest that a population of antigen-specific antibodies can have transmission-blocking activity by blocking the biological function of targeted proteins, such as blocking the fertilization of gametes, while other antibodies are involved in the complement-mediated lysis of gametes within the mosquitoes [72]. Neutralizing antibodies against gametocyte and ookinete surface proteins could block the parasite fertilization, zygote transformation and subsequent traversal of the mosquito midgut, all critical steps in the *Plasmodium* life cycle [78]. In contrast, other studies showed that antibodies against *P. falciparum*, obtained from immunized or naturally exposed hosts and fed to infected *An. stephensi* mosquitoes, were detected to bind sporozoites in the hemolymph, but did not reduce sporozoite infection in the salivary glands [79, 80]. Proteins expressed by pathogens preferentially during transmission to the tick (e.g. OspA and BBA52) or mosquito (e.g. Pfs25) vectors should not elicit specific antibodies in the vertebrate hosts. For example, malaria-exposed individuals do not mount Pfs25-specific immune responses [81]. The absence of immune pressure on surface proteins expressed by the pathogen during infection in the vector has been associated with remarkable sequence conservation [78], which further supports the use of these antigens as vaccine candidates.

## Interactions of host immune components with symbionts and commensal microbes within vectors

Functional host antibodies have been shown to interact with symbionts in *R. prolixus* [82] and *Glossina morsitans* [83] as well as with bacterial microbiota in mosquitoes [84] and ticks [25, 26]. *Rhodnius prolixus* fed exclusively on blood from rabbits immunized against *Rhodoccocus rhodnii* have developmental alterations such as prolonged molting times, incomplete development and malformed limbs [82]. Feeding of *R. prolixus* larvae on hosts immunized against their symbiont produces retardation of the symbiont growth [82]. Developmental alterations observed in *R. prolixus* fed on *R. rhodnii*-immunized animals were similar to those described in aposymbiotic triatomines (sterile-raised and germ-free insects that lack *R. rhodnii*) [85]. Interestingly, in addition to *R. rhodnii*-specific antibodies, it was observed that recently fed bugs contained numerous symbiont cells within host macrophages found in *R. prolixus* guts [85]. Accordingly, cell-mediated immunity, especially primed macrophages, was proposed as playing a fundamental role in the reduction of *R. rhodnii* levels within *R. prolixus*. Similar results were obtained by Nogge [83] who found that tsetse flies fed on rabbits immunized with symbionts became aposymbiotic, and their fecundity decreased drastically while their longevity was not affected. Furthermore, a significant number of flies maintained on rabbits immunized with gut bacteria had permanently laterally extended wings [86]. The extended wings are probably due to weakness of thoracic flight muscles. Those wings were paralyzed, which impaired flying and therefore trypanosomes transmission, and the mortality rate was much higher in flies that fed on immunized rabbits [86].

Another study addressed the question of whether antibodies against midgut microbiota bacteria could impair *Plasmodium* spp. life cycle within *A. gambiae* mosquitoes [84]. To this aim, rabbits were immunized against whole midgut lysates of *A. gambiae*. Immune sera contained IgG specific to midgut lysates and two gram-negative bacterial species, *Pseudomonas aeruginosa* and *Cedecea lapagei*, isolated from the mosquito midguts [84]. A significantly higher prevalence of *P. falciparum* oocysts was found in mosquitoes fed on gametocyte cultures mixed with the immune sera, while the same immune sera did not affect *Plasmodium yoelii* oocyst development [84]. The differential effect observed in the two *Plasmodium* species could be explained by differences in their life cycle in relation to the expansion of midgut bacterial populations after blood feeding. The authors suggested that the midgut microbiota probably exert a greater influence on the ookinetes of late-developing species such as *P. falciparum* compared to early-developing species such as *P. yoelii*. Notably, despite antibacterial IgG bound *P. aeruginosa* and *C. lapagei*, the immune sera did not inhibit the growth of these bacteria in vitro [84].

More recently, anti-microbiota vaccines were designed to target specific taxa within tick microbiota [25, 26]. The genus *Escherichia*-*Shigella*, identified as central in the tick microbial communities, was targeted with host antibodies [25]. Immunization of mice against live *E. coli* induced high levels of *E. coli*-specific IgM and IgG that were negatively correlated with the abundance of *Escherichia*-*Shigella* in tick microbiota [26]. The weight of nymph ticks that fed on *E. coli*-immunized mice increased significantly compared with ticks fed on mock-immunized mice. Immunization with *E. coli* was associated with increased engorgement weight of *Ixodes ricinus* nymphs [25, 26]. Strong and specific immune reaction of mouse IgM against *E. coli* was confirmed by immunofluorescence, while the reaction of anti-*E. coli* IgG against the bacteria was comparatively less intense [26]. Furthermore, high mortality was observed in ticks fed on mice with high levels of IgM and IgG targeting the carbohydrate α-Gal, broadly present in the tick microbiota [25].

## Using host antibodies for microbiome manipulation: the forgotten strategy for blocking pathogen transmission?

**Fig. 2:**
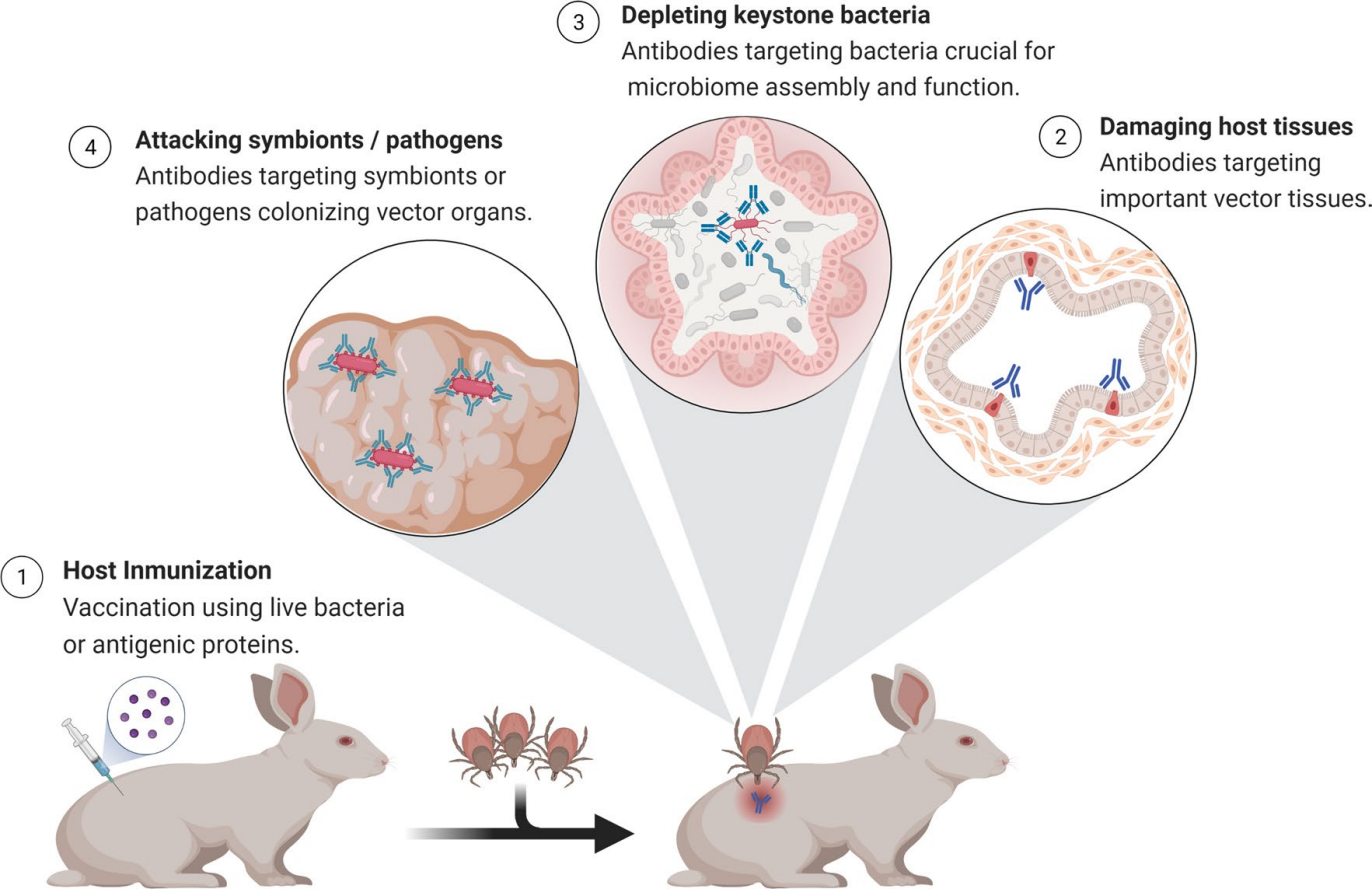
Multiple functionalities of host antibodies within ticks. Antibodies induced by immunization against specific cells or antigens have multiple functionalities within ticks. These host molecules can target symbionts, commensal bacteria and tissues. Created with BioRender.com

Host antibodies have multiple functionalities within ticks, as they can target symbionts, commensal bacteria and tissues (Fig. 2). However, many research questions remain open such as: Can immunity against microbiota bacteria modulate the structure and function of microbial communities within the vectors? Can host immunity be used as a tool for microbiome manipulation? Can immunity against a single bacteria species trigger cascading ecological effects on the whole microbiome with consequences for vector-pathogen interactions and pathogen transmission to the host as well as host life history traits? Answering those questions requires further research into the impact of host immunity on vector-pathogen-microbiome multipartite interactions.

Recently, our team used a live bacteria vaccine as a tool to manipulate the tick microbiome. Indeed, vaccination against the keystone bacteria *E. coli* reduced bacterial diversity of the tick microbiome compared to unfed ticks [26]. Co-occurrence network analysis showed that the immunization with *E. coli* reduced the number of edges and thus the number of interactions among the bacterial taxa of the tick microbiome compared to the control group. While the proportions of positive or negative interactions as well as the network diameter and modularity were similar between the control group and the *E. coli* group, the number of modules increased in the microbiome of ticks fed on *E. coli*-immunized mice compared to the control group [26]. Furthermore, local connectivity analysis showed that the *E. coli* vaccine drastically reduced the direct interactions of the taxon *Escherichia-Shigella* with other taxa in the microbiome of ticks fed on *E. coli*-immunized mice compared to ticks fed on mock-immunized mice [26]. The eigenvector centrality value of *Escherichia-Shigella* also decreased in the networks inferred from ticks fed on *E. coli*-immunized mice compared to the control group. At the functional levels, the abundance of several pathways changed significantly between the control and *E. coli* group. Notably, the relative abundance of one of these pathways (l-lysine fermentation to acetate and butanoate pathway) was found exclusively in ticks fed on *E. coli*-immunized mice [26]. These results showed that an anti-microbiota vaccine against a keystone bacterium can modulate the tick microbiome not only at the taxonomic but also at the bacterial community level by shifting the structure, interactions and functional profile of microbial communities within the vector suggesting that anti-microbiota vaccine can be a suitable tool for specific manipulation of the vector microbiome.

## Conclusions

**Fig. 3:**
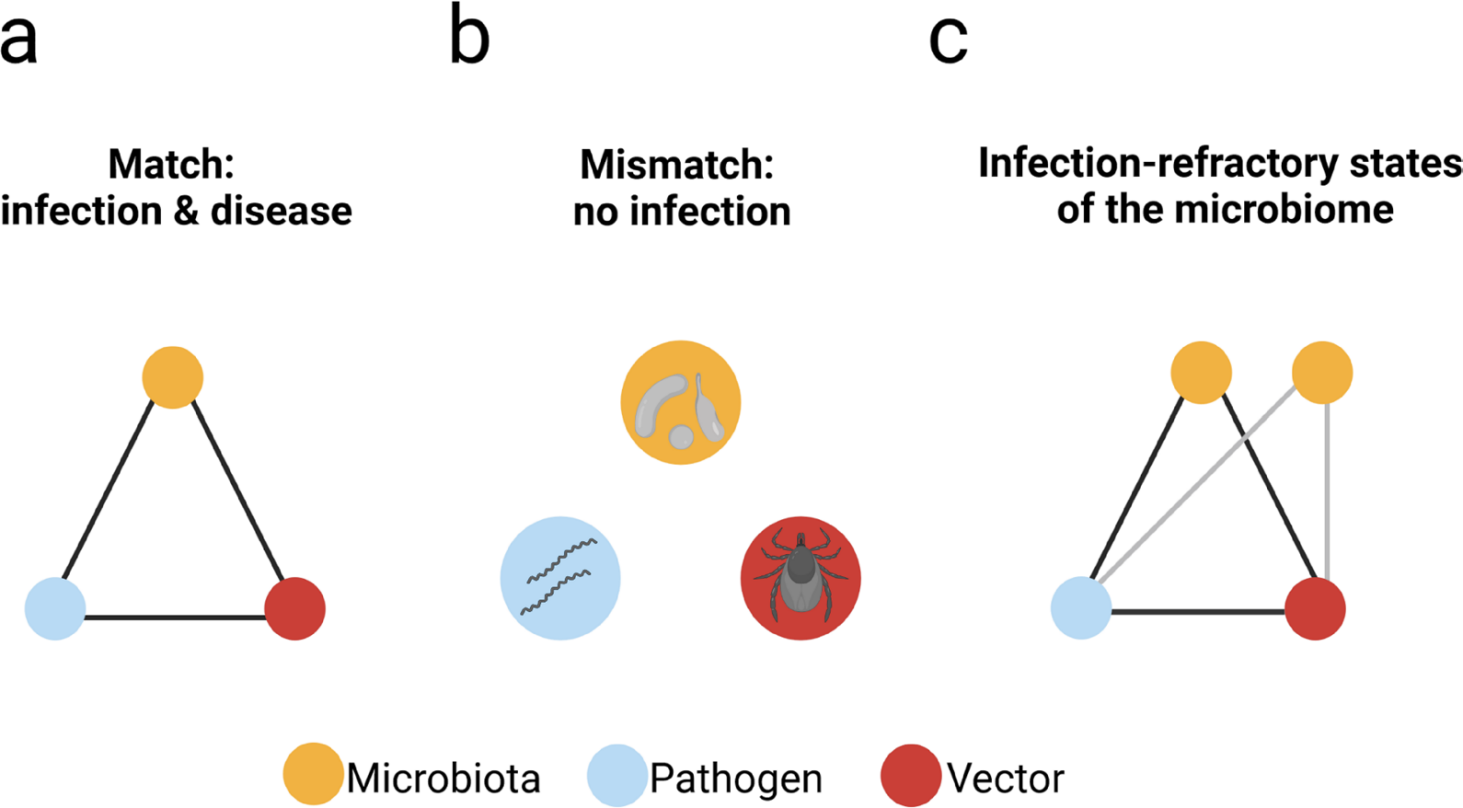
Inducing infection-refractory states in vector microbiome. **a** Chains of infection of vector-borne diseases such as those transmitted by ticks involve matching (black lines) among competent vectors (red circle), infectious pathogens (blue circle) and an infection-compatible microbiome (yellow circle). **b** Mismatches between at least two of the components can result in an impaired ability of the vector to transmit pathogens. **c** Microbiome manipulation using anti-microbiota vaccines that elicit microbiota specific antibodies can be used to induce infection-refractory states (gray lines) in vector microbiome in which microbiome manipulation results in transmission blocking despite matching between pathogen and vector genetics (black line). Created with BioRender.com

Hematophagous ectoparasites ingest large amounts of blood containing host antibodies, complement proteins and immune cells. These immune components retain their immune functions within the midguts of arthropod vectors. This offers the unique opportunity of targeting vector bacterial microbiota with specific antibodies to disrupt the vector-pathogen-microbiota homeostasis. Effective chains of infection of vector-borne pathogens involve competent vectors, infective pathogens and an infection-compatible microbiome (Fig. 3a). Mismatch of at least one of the components (e.g. pathogen genetics, vector genetics or microbiota composition) can result in an impaired ability of the vector to transmit pathogens (Fig. 3b). For example, population replacement (a strategy based on reducing the vector competence for pathogens by genetically modifying insects that no longer transmit pathogens) is one of the strategies used for vector and/or pathogen control [20, 87]. As revised here, there is strong evidence showing that alterations in the vector midgut microbiomes affect pathogen transmission and infection. Therefore, deviations from infection-compatible microbiomes could block transmission and disease development (Fig. 3c). Anti-microbiota vaccines can be used as a microbiome manipulation tool for the induction of infection-refractory states in the vector microbiome (Fig. 3c). A current limitation of this approach is that most bacteria in the vector microbiota are unable to grow in standard laboratory media, which makes isolating unculturable bacteria a major challenge in current microbiological research. Shotgun metagenomics could be applied to the mapping of antigenic proteins in the bacterial microbiota of vectors. Identified antigenic proteins from bacterial candidates could be used as an alternative to live bacterial vaccines used in current anti-microbiota vaccination approaches [25, 26]. Understanding specific traits, such as variance in microbiota dynamics at individual and population levels, and whether that relates to vertebrate host immune system-microbiota interactions will be of great importance for future research. Likewise, new protocols now make it possible to manipulate the microbiota of arthropod vectors to generate axenic and gnotobiotic individuals (associated with specific microorganisms [88]). Such development could help to validate such an approach based on the use of host antibodies for microbiota manipulation.

## Data Availability

No new data were created or analyzed in this study. Data sharing is not applicable to this article.

## References

[1] de Fuente J, Antunes S, Bonnet S, Cabezas-Cruz A, Domingos AG, Estrada-Peña A, Johnson N, Kocan KM, Mansfield KL, Nijhof AM, Papa A, Rudenko N, Villar M, Alberdi P, Torina A, Ayllón N, Vancova M, Golovchenko M, Grubhoffer L, Caracappa S, Fooks AR, Gortazar C, Rego ROM (2017). Tick-pathogen interactions and vector competence: identification of molecular drivers for tick-borne diseases. Front Cell Infect Microbiol.

[2] Lee H, Halverson S, Ezinwa N (2018). Mosquito-Borne Diseases. Primary Care: Clinics in Office Practice.

[3] Serafim TD, Coutinho-Abreu IV, Dey R, Kissinger R, Valenzuela JG, Oliveira F, Kamhawi S (2021). Leishmaniasis: The Act of Transmission. Trends Parasitol.

[4] Hamzaoui BE, Zurita A, Cutillas C, Parola P (2020). Fleas and Flea-Borne Diseases of North Africa. Acta Trop.

[5] Sonenshine DE, Simo L. Biology and Molecular Biology of *Ixodes scapularis*. In: Lyme Disease and Relapsing Fever Spirochetes: Genomics, Molecular Biology, Host Interactions and Disease Pathogenesis; Caister Academic Press, 2021. 10.21775/9781913652616.12.

[6] Gabrieli P, Caccia S, Varotto-Boccazzi I, Arnoldi I, Barbieri G, Comandatore F, Epis S (2021). Mosquito trilogy: microbiota, immunity and pathogens, and their implications for the control of disease transmission. Front Microbiol.

[7] Narasimhan S, Fikrig E (2015). Tick microbiome: the force within. Trends Parasitol.

[8] Wu-Chuang A, Hodžić A, Mateos-Hernández L, Estrada-Peña A, Obregon D, Cabezas-Cruz A (2021). Current debates and advances in tick microbiome research. Curr Res Parasitol Vector-Borne Diseases.

[9] Duron O, Gottlieb Y (2020). Convergence of nutritional symbioses in obligate blood feeders. Trends Parasitol.

[10] Zhong Z, Zhong T, Peng Y, Zhou X, Wang Z, Tang H, Wang J (2021). Symbiont-regulated serotonin biosynthesis modulates tick feeding activity. Cell Host Microbe.

[11] Obregón D, Bard E, Abrial D, Estrada-Peña A, Cabezas-Cruz A (2019). Sex-specific linkages between taxonomic and functional profiles of tick gut microbiomes. Front Cell Infect Microbiol.

[12] Wang Y, Hoon Eum J, Harrison R, Valzania L, Yang X, Johnson J, Huck D, Brown M, Strand M (2021). Riboflavin instability is a key factor underlying the requirement of a gut microbiota for mosquito development. Proc Natl Acad Sci.

[13] Michalkova V, Benoit JB, Weiss BL, Attardo GM, Aksoy S (2014). Vitamin B6 generated by obligate symbionts is critical for maintaining proline homeostasis and fecundity in tsetse flies. Appl Environ Microbiol.

[14] Estrada-Peña A, Cabezas-Cruz A, Obregón D (2020). Behind taxonomic variability: the functional redundancy in the tick microbiome. Microorganisms.

[15] Estrada-Peña A, Cabezas-Cruz A, Obregón D (2020). Resistance of tick gut microbiome to anti-tick vaccines, pathogen infection and antimicrobial peptides. Pathogens.

[16] Romoli O, Schönbeck JC, Hapfelmeier S, Gendrin M (2021). Production of germ-free mosquitoes via transient colonisation allows stage-specific investigation of host–microbiota interactions. Nat Commun.

[17] Hajkazemian M, Bossé C, Mozūraitis R, Emami SN (2021). Battleground midgut: The cost to the mosquito for hosting the malaria parasite. Biol Cell.

[18] Wang M, An Y, Gao L, Dong S, Zhou X, Feng Y, Wang P, Dimopoulos G, Tang H, Wang J (2021). Glucose-mediated proliferation of a gut commensal bacterium promotes *Plasmodium* infection by increasing mosquito midgut pH. Cell Rep.

[19] Bando H, Okado K, Guelbeogo WM, Badolo A, Aonuma H, Nelson B, Fukumoto S, Xuan X, Sagnon N, Kanuka H (2013). Intra-specific diversity of *Serratia marcescens* in *Anopheles* Mosquito midgut defines plasmodium transmission capacity. Sci Rep.

[20] Shaw WR, Catteruccia F (2019). Vector biology meets disease control: using basic research to fight vector-borne diseases. Nat Microbiol.

[21] Gendrin M, Rodgers FH, Yerbanga RS, Ouédraogo JB, Basáñez M-G, Cohuet A, Christophides GK (2015). Antibiotics in ingested human blood affect the mosquito microbiota and capacity to transmit malaria. Nat Commun.

[22] Gendrin M, Yerbanga RS, Ouedraogo JB, Lefèvre T, Cohuet A, Christophides GK (2016). Differential effects of azithromycin, doxycycline, and cotrimoxazole in ingested blood on the vectorial capacity of malaria mosquitoes. Open Forum Infect Dis.

[23] Gall CA, Reif KE, Scoles GA, Mason KL, Mousel M, Noh SM, Brayton KA (2016). The bacterial microbiome of *Dermacentor andersoni* ticks influences pathogen susceptibility. ISME J.

[24] Narasimhan S, Swei A, Abouneameh S, Pal U, Pedra JHF, Fikrig E (2021). Grappling with the tick microbiome. Trends Parasitol.

[25] Mateos-Hernández L, Obregón D, Maye J, Borneres J, Versille N, de la Fuente J, Estrada-Peña A, Hodžić A, Šimo L, Cabezas-Cruz A (2020). Anti-tick microbiota vaccine impacts *Ixodes ricinus* performance during feeding. Vaccines.

[26] Mateos-Hernández L, Obregón D, Wu-Chuang A, Maye J, Bornères J, Versillé N, de la Fuente J, Díaz-Sánchez S, Bermúdez-Humarán LG, Torres-Maravilla E, Estrada-Peña A, Hodžić A, Šimo L, Cabezas-Cruz A (2021). Anti-microbiota vaccines modulate the tick microbiome in a taxon-specific manner. Front Immunol.

[27] Wu-Chuang A, Obregon D, Mateos-Hernández L, Cabezas-Cruz A (2021). Anti-tick microbiota vaccines: how can this actually work?. Biologia.

[28] Narasimhan S, Rajeevan N, Liu L, Zhao YO, Heisig J, Pan J, Eppler-Epstein R, DePonte K, Fish D, Fikrig E (2014). Gut Microbiota of the Tick Vector *Ixodes scapularis* Modulate colonization of the lyme disease spirochete. Cell Host Microbe.

[29] Narasimhan S, Schuijt TJ, Abraham NM, Rajeevan N, Coumou J, Graham M, Robson A, Wu M-J, Daffre S, Hovius JW, Fikrig E (2017). Modulation of the tick gut milieu by a secreted tick protein favors *Borrelia burgdorferi* Colonization. Nat Commun.

[30] Abraham NM, Liu L, Jutras BL, Yadav AK, Narasimhan S, Gopalakrishnan V, Ansari JM, Jefferson KK, Cava F, Jacobs-Wagner C, Fikrig E (2017). Pathogen-mediated manipulation of arthropod microbiota to promote infection. Proc Natl Acad Sci USA.

[31] Heisig M, Abraham NM, Liu L, Neelakanta G, Mattessich S, Sultana H, Shang Z, Ansari JM, Killiam C, Walker W, Cooley L, Flavell RA, Agaisse H, Fikrig E (2014). Antivirulence properties of an antifreeze protein. Cell Rep.

[32] Brinkerhoff RJ, Clark C, Ocasio K, Gauthier DT, Hynes WL (2020). Factors Affecting the Microbiome of *Ixodes scapularis* and *Amblyomma americanum*. PLoS ONE.

[33] Chauhan G, McClure J, Hekman J, Marsh PW, Bailey JA, Daniels RF, Genereux DP, Karlsson EK (2020). Combining citizen science and genomics to investigate tick, pathogen, and commensal microbiome at single-tick resolution. Front Genet.

[34] Huang W, Wang S, Jacobs-Lorena M (2020). Use of microbiota to fight mosquito-borne disease. Front Genet.

[35] Tchioffo MT, Boissière A, Churcher TS, Abate L, Gimonneau G, Nsango SE, Awono-Ambéné PH, Christen R, Berry A, Morlais I (2013). Modulation of malaria infection in *Anopheles gambiae* mosquitoes exposed to natural midgut bacteria. PLoS ONE.

[36] Cirimotich CM, Ramirez JL, Dimopoulos G (2011). Native microbiota shape insect vector competence for human pathogens. Cell Host Microbe.

[37] Wu P, Sun P, Nie K, Zhu Y, Shi M, Xiao C, Liu H, Liu Q, Zhao T, Chen X, Zhou H, Wang P, Cheng G (2019). A gut commensal bacterium promotes mosquito permissiveness to arboviruses. Cell Host Microbe.

[38] Dong Y, Manfredini F, Dimopoulos G (2009). Implication of the mosquito midgut microbiota in the defense against malaria parasites. PLoS Pathog.

[39] Meister S, Agianian B, Turlure F, Relógio A, Morlais I, Kafatos FC, Christophides GK (2009). *Anopheles gambiae* PGRPLC-mediated defense against bacteria modulates infections with malaria parasites. PLoS Pathog.

[40] Garver LS, Dong Y, Dimopoulos G (2009). Caspar controls resistance to *Plasmodium falciparum* in diverse anopheline species. PLoS Pathog.

[41] Azambuja P, Feder D, Garcia ES (2004). Isolation of serratia marcescens in the midgut of rhodnius prolixus: impact on the establishment of the parasite *Trypanosoma cruzi* in the Vector. Exp Parasitol.

[42] Fieck A, Hurwitz I, Kang AS, Durvasula R (2010). Trypanosoma Cruzi: synergistic cytotoxicity of multiple amphipathic anti-microbial peptides to *T. cruzi* and potential bacterial hosts. Exp Parasitol.

[43] Frentiu FD, Zakir T, Walker T, Popovici J, Pyke AT, van den Hurk A, McGraw EA, O’Neill SL (2014). Limited h *Aedes aegypti* mosquitoes infected with Wolbachia. PLoS Negl Trop Dis.

[44] Schmidt TL, Barton NH, Rašić G, Turley AP, Montgomery BL, Iturbe-Ormaetxe I, Cook PE, Ryan PA, Ritchie SA, Hoffmann AA, O’Neill SL, Turelli M (2017). Local Introduction and Heterogeneous Spatial Spread of Dengue-Suppressing *Wolbachia* through an Urban Population of *Aedes aegypti*. PLoS Biol.

[45] Beier MS, Pumpuni CB, Beier JC, Davis JR (1994). Effects of para-aminobenzoic acid, insulin, and gentamicin on *Plasmodium falciparum* Development in Anopheline Mosquitoes (Diptera: Culicidae). J Med Entomol.

[46] Moreira LA, Iturbe-Ormaetxe I, Jeffery JA, Lu G, Pyke AT, Hedges LM, Rocha BC, Hall-Mendelin S, Day A, Riegler M, Hugo LE, Johnson KN, Kay BH, McGraw EA, van den Hurk AF, Ryan PA, O’Neill SL (2009). A *Wolbachia* Symbiont in *Aedes aegypti* limits infection with dengue, chikungunya, and plasmodium. Cell.

[47] Walker T, Johnson PH, Moreira LA, Iturbe-Ormaetxe I, Frentiu FD, McMeniman CJ, Leong YS, Dong Y, Axford J, Kriesner P, Lloyd AL, Ritchie SA, O’Neill SL, Hoffmann AA (2011). The WMel *Wolbachia* Strain Blocks Dengue and Invades Caged *Aedes aegypti* Populations. Nature.

[48] Landmann F, Cossart P, Craig RR, Sansonetti P (2019). The Wolbachia Endosymbionts. Am Soc Microbiol.

[49] Dutra HLC, Rocha MN, Dias FBS, Mansur SB, Caragata EP, Moreira LA (2016). Wolbachia blocks currently circulating zika virus isolates in Brazilian *Aedes aegypti* Mosquitoes. Cell Host Microbe.

[50] Ackerman S, Clare FB, McGill TW, Sonenshine DE (1981). Passage of host serum components, including antibody, across the digestive tract of *Dermacentor variabilis* (Say). J Parasitol.

[51] Ben-Yakir D, Fox CJ, Homer JT, Barker RW (1987). Quantification of host immunoglobulin in the hemolymph of ticks. J Parasitol.

[52] Wang H, Nuttall PA (1994). Excretion of host immunoglobulin in tick saliva and detection of igg-binding proteins in tick haemolymph and salivary glands. Parasitology.

[53] Willadsen P (1997). Novel vaccines for ectoparasites. Vet Parasitol.

[54] Rathinavelu S, Broadwater A, de Silva AM (2003). Does Host Complement Kill *Borrelia burgdorferi* within Ticks?. Infect Immun.

[55] Galay RL, Matsuo T, Hernandez EP, Talactac MR, Kusakisako K, Umemiya-Shirafuji R, Mochizuki M, Fujisaki K, Tanaka T (2018). Immunofluorescent detection in the ovary of host antibodies against a secretory ferritin injected into Female *Haemaphysalis longicornis* Ticks. Parasitol Int.

[56] Vaughan JA (2002). Kinetics of ingested host immunoglobulin g in hemolymph and whole body homogenates during nymphal development of *Dermacentor variabilis* and *Ixodes scapularis* Ticks (Acari: Ixodidae). Exp Appl Acarol.

[57] Chinzei Y, Minoura H (1987). Host Immunoglobulin G Titre and Antibody Activity in Haemolymph of the Tick *Ornithodoros moubata*. Med Vet Entomol.

[58] Hatfield PR (1988). Detection and localization of antibody ingested with a mosquito bloodmeal. Med Vet Entomol.

[59] Lackie AM, Gavin S (1989). Uptake and persistence of ingested antibody in the mosquito *Anopheles stephensi*. Med Vet Entomol.

[60] Tesh RB, Chen W-R, Catuccio D (1988). Survival of Albumin, IgG, IgM, and Complement (C3) in human blood after ingestion by *Aedes albopictus* and *Phlebotomus papatasi*. Am J Trop Med Hyg.

[61] Saab NAA, Nascimento AAS, Queiroz DC, da Cunha IGM, Filho AAP, D’Ávila Pessoa GC, Koerich LB, Pereira MH, SantAnna MRV, Araújo RN, Gontijo NF (2020). How *Lutzomyia longipalpis* Deals with the Complement System Present in the Ingested Blood: The Role of Soluble Inhibitors and the Adsorption of Factor H by Midgut. J Insect Physiol.

[62] Nogge G, Giannetti M (1980). Specific Antibodies: A Potential Insecticide. Science.

[63] Vaughan JA, Azad AF (1988). Passage of host immunoglobulin G from blood meal into hemolymph of selected mosquito species (Diptera: Culicidae). J Med Entomol.

[64] Margos G, Navarette S, Butcher G, Davies A, Willers C, Sinden RE, Lachmann PJ (2001). Interaction between Host Complement and Mosquito-Midgut-Stage *Plasmodium berghei*. Infect Immun.

[65] Gough JM, Kemp DH (1993). Localization of a Low Abundance Membrane Protein (Bm86) on the Gut Cells of the Cattle Tick *Boophilus microplus* by Immunogold Labeling. J Parasitol.

[66] de la Fuente J, Moreno-Cid JA, Canales M, Villar M, de la Lastra JMP, Kocan KM, Galindo RC, Almazán C, Blouin EF (2011). Targeting arthropod subolesin/akirin for the development of a universal vaccine for control of vector infestations and pathogen transmission. Vet Parasitol.

[67] Rodríguez-Mallon A, Encinosa PE, Méndez-Pérez L, Bello Y, Rodríguez Fernández R, Garay H, Cabrales A, Méndez L, Borroto C, Estrada MP (2015). High Efficacy of a 20 amino Acid Peptide of the Acidic Ribosomal Protein P0 against the Cattle Tick. Rhipicephalus Microplus Ticks Tick-borne Dis.

[68] Rodríguez-Mallon A, Fernández E, Encinosa PE, Bello Y, Méndez-Pérez L, Ruiz LC, Pérez D, González M, Garay H, Reyes O, Méndez L, Estrada MP (2012). A novel tick antigen shows high vaccine efficacy against the dog tick *Rhipicephalus sanguineus*. Vaccine.

[69] Meyers JI, Gray M, Foy BD (2015). Mosquitocidal Properties of IgG Targeting the Glutamate-Gated Chloride Channel in Three Mosquito Disease Vectors (Diptera: Culicidae). J Exp Biol.

[70] Artigas-Jerónimo S, Villar M, Cabezas-Cruz A, Valdés JJ, Estrada-Peña A, Alberdi P, de la Fuente J (2018). Functional evolution of subolesin/akirin. Front Physiol.

[71] Kumar M, Kaur S, Kariu T, Yang X, Bossis I, Anderson JF, Pal U (2011). *Borrelia burgdorferi* BBA52 is a potential target for transmission blocking lyme disease vaccine. Vaccine.

[72] Tachibana M, Wu Y, Iriko H, Muratova O, MacDonald NJ, Sattabongkot J, Takeo S, Otsuki H, Torii M, Tsuboi T (2011). N-Terminal Prodomain of Pfs230 synthesized using a cell-free system is sufficient to induce complement-dependent malaria transmission-blocking activity. Clin Vaccine Immunol.

[73] Chowdhury DR, Angov E, Kariuki T, Kumar N (2009). A potent malaria transmission blocking vaccine based on codon harmonized full length Pfs48/45 expressed in *Escherichia coli*. PLoS ONE.

[74] Kapulu MC, Da DF, Miura K, Li Y, Blagborough AM, Churcher TS, Nikolaeva D, Williams AR, Goodman AL, Sangare I, Turner AV, Cottingham MG, Nicosia A, Straschil U, Tsuboi T, Gilbert SC, Long CA, Sinden RE, Draper SJ, Hill AVS, Cohuet A, Biswas S (2015). Comparative assessment of transmission-blocking vaccine candidates against *Plasmodium falciparum*. Sci Rep.

[75] de Silva AM, Telford SR, Brunet LR, Barthold SW, Fikrig E (1996). Borrelia Burgdorferi OspA is an arthropod-specific transmission-blocking lyme disease vaccine. J Exp Med.

[76] Gipson CL, de Silva AM (2005). Interactions of OspA Monoclonal Antibody C378 with *Borrelia burgdorferi* within Ticks. Infect Immun.

[77] Sinden RE (2017). Developing transmission-blocking strategies for malaria control. PLoS Pathog.

[78] Matuschewski K, Mueller A-K (2007). Vaccines against malaria - an update: anti-malaria vaccine development. FEBS J.

[79] Vaughan JA, Do Rosario V, Leland P, Adjepong A, Light J, Woollett GR, Hollingdale MR, Azad AF (1988). Plasmodium falciparum: ingested anti-sporozoite antibodies affect sporogony in *Anopheles stephensi* mosquitoes. Exp Parasitol.

[80] Beier JC, Oster CN, Koros JK, Onyango FK, Githeko AK, Rowton E, Koech DK, Roberts CR (1989). Effect of human circumsporozoite antibodies in *Plasmodium*-infected *Anopheles* (Diptera: Culicidae). J Med Entomol.

[81] Carter R, Graves PM, Quakyi IA, Good MF (1989). Restricted or absent immune responses in human populations to *Plasmodium falciparum* Gamete antigens that are targets of malaria transmission-blocking antibodies. J Exp Med.

[82] Ben-Yakir D (1987). Growth retardation of *Rhodnius prolixus* symbionts by immunizing host against *Nocardia (Rhodococcus) Rhodnii*. J Insect Physiol.

[83] Nogge G (1978). Aposymbiotic Tsetse Flies, *Glossina morsitans morsitans* obtained by feeding on rabbits immunized specifically with symbionts. J Insect Physiol.

[84] Noden BH, Vaughan JA, Pumpuni CB, Beier JC (2011). Mosquito Ingestion of Antibodies against Mosquito Midgut Microbiota Improves Conversion of Ookinetes to Oocysts for Plasmodium falciparum, but Not P. yoelii. Parasitol Int.

[85] Salcedo-Porras N, Umaña-Diaz C, de Oliveira R, Lowenberger C (2020). The role of bacterial symbionts in triatomines: an evolutionary perspective. Microorganisms.

[86] Kaaya GP, Alemu P (1984). Further observations on survival and fertility of *Glossina morsitans morsitans* maintained on immunized rabbits. Int J Trop Insect Sci.

[87] Burt A (2003). Site-specific selfish genes as tools for the control and genetic engineering of natural populations. Proc R Soc Lond B.

[88] Steven B, Hyde J, LaReau JC, Brackney DE (2021). The axenic and gnotobiotic mosquito: emerging models for microbiome host interactions. Front Microbiol.

